# Effect of estradiol on proliferation and differentiation of side population stem/progenitor cells from murine endometrium

**DOI:** 10.1186/1477-7827-9-103

**Published:** 2011-07-29

**Authors:** Jing Xu, Fei-Fei Hu, Yu-Gui Cui, Jian Luo, Chun-Yan Jiang, Li Gao, Xiao-Qiao Qian, Yun-Dong Mao, Jia-Yin Liu

**Affiliations:** 1 State Key Laboratory of Reproductive Medicine, Center of Clinical Reproductive Medicine, First Affiliated Hospital, Nanjing Medical University, Nanjing 210029, China; 2 Zhenjiang Maternity and Child Health Care Hospital, Zhenjiang, 212000, China; 3 Department of Gynecology and Obstetrics, Second Affiliated Hospital, Nanjing Medical University, Nanjing 210003, China

## Abstract

**Background:**

In our previous study, endometrium side population cells (SP cells) were isolated from postpartum murine uterus, and characterized by a heterogeneous population of stem/progenitor cells. In this study, we investigated the effect of estrogen on the proliferation and differentiation of SP cells.

**Methods:**

SP and non-SP cells of postpartum murine endometrium were isolated by DNA dye Hoechst 33342. The expression of estrogen receptor 1 (ESR1) was measured by reverse transcription polymerase chain reaction (RT-PCR), Real-time PCR, Western blot, immunofluorescence and immunohistochemistry. The proliferation and differentiation of SP cells treated with different concentrations [10(-8) M-10(-6) M] of estradiol (E2) and E2+ ICI182780 (Faslodex, inhibitor of ESR1) were measured by 3-(4, 5-dimethylthiazoly1-2)-2,5-diphenyltetrazolium bromide(MTT) and clonogenic assays.

**Results:**

(1) SP cells expressed ESR1 at a higher level than non-SP cells. (2) The level of E2 in the serum and the expression of ESR1 in the uterus of postpartum murine changed in the same manner with the ratio of SP cells to total uterus cells at a different postpartum time point. ESR1, as ABCG2 is also predominantly located in the stroma and the glandular epithelium of the uterus. (3) 10(-6) M E2 notably promoted the proliferation of SP cells after treatment for 24 h. This effect could be inhibited by ICI182780. E2 at the concentration of 10(-7) M or 10(-8) M was sent to impair the large cloning efficiency (CE) of SP cells.

**Conclusions:**

The effect of estrogen on the proliferation and differentiation of endometrium SP cells via ESR1 was observed and it was in a concentration dependent fashion. Clearly, more work is needed to understand the *in vivo *effect of E2 at the physiological concentration on the differentiation of SP cells.

## Background

It has been proposed that human endometrium contain a population of stem cells which are responsible for their remarkable regenerative ability [1,2]. Side population cells (SP cells) have been shown in many adult tissues, and the phenotypes of SP cells might represent common molecular features for a wide variety of stem cells [1-3]. In a previous study, SP cells were isolated from the endometrium of postpartum murine uterus and these SP cells were characterized by a heterogeneous population of stem/progenitor cells [3]. Estrogen is an important hormone for repairing postpartum uterus endometrium repairing. Estrogen receptor (ESR) has two isoforms: ESR1 and ESR2. Although both ESR1 and ESR2 are present in the endometrium, ESR1 seems to be the primary mediator of the estrogenic action in these tissues [4]. Some investigators found that ESR1 amplification and over-expression is likely to have a growth stimulatory effect on endometrium-derived cancer cells [5]. It is important to know how SP cells participate in the repair of cyclical and postpartum endometrium, and the effect of estrogen (via ESR1) in this procedure. Meanwhile, research on the proliferation and differentiation of endometrium SP cells, as well as the effect of steroid hormones, will add knowledge to our understanding of pathophysiology of endometriosis.

The objectives of the present study were: 1) To evaluate the potential of the *in vivo *effect of estrogen on the proliferation and differentiation of SP cells during endometrium repairing by investigation of the serum estradiol level and the expression of ESR1 in murine uterus at different postpartum stages. 2) To observe directly the *in vitro *effect of estradiol on the proliferation and differentiation of cultured SP cells treated with different concentrations of E_2 _and ICI182780 (inhibitor of ESR1).

## Methods

### Animals

Female ICR mice [Institute of Cancer Research (ICR)], aged 6-8 weeks, were used. ICR mice were purchased from Model Animal Research Center of Nanjing University (Nanjing, China). Sixty mice were divided into six groups based on their postpartum day (Day 1, 7, 14, 18, 21, 28) to detect serum estradiol (E_2_) level and the expression of estrogen receptor 1 (ESR1) in postpartum endometrium. Another 60 ICR mice were used at postpartum Day 18 to isolate endometrium side population (SP) cells. Animal studies were conducted according to the protocols approved by the Animal Care and Use Committee of Nanjing Medical University.

### Cell preparation

Endometrium SP cells were isolated and cultured using pancreatic enzyme, collagenase, as well as mechanical separation [3]. Cells were suspended at a concentration of 1 × 10^6 ^cells/ml and were then incubated in 5 μg/ml Hoechst 33342 dye (Sigma-Aldrich, St. Louis, MO). Suspensions were analyzed and sorted using a FACS Vantage SE cell sorter (Becton Dickinson, Franklin Lakes, NJ) with a 350 nm UV diode laser. Hoechst 33342 fluorescence was measured at both 402 - 446 nm for Hoechst blue and 640 nm for Hoechst red.

### Immunocytochemistry

The freshly sorted SP cells were collected and re-suspended to a final concentration of 1 × 10^6^/ml. An aliquot of 0.2 ml of the suspension was used for each cell smear. Cells were cytospun onto plus-coated slides, air dried, and fixed in acetone for 10 min at 4°C. The sections were incubated with anti- ESR1 pAb (1:50 dilution, Santa Cruz, CA) for 24 hours and then with FITC secondary antibodies. Nuclear staining was performed with 5 μg/ml Hoechst 33342. The percentage of positive expression of ESR1 was calculated by counting positive cells in every 400 cells from three independent experiments.

Five-micrometer-thick coronal sections (paraffin-embedded) of postpartum mouse uterus were exposed to citrate buffer (0.01 M, pH 6.0) and heated in a microwave oven for 10 minutes for antigen retrieve. The sections were treated with 0.3% hydrogen peroxide in methyl alcohol for 20 minutes and blocked with 10% normal goat serum (Zhongshan Biotechnology Co. Ltd., Beijing, China) for 2 hours. It was allowed to react for 12 hours with the primary rabbit anti-ESR1 antibody (1:50 dilution, Santa Cruz, CA) or normal rabbit IgG (1:100 dilution, Santa Cruz, CA) as a negative control. Sections were then incubated with horseradish peroxidase (HRP)-conjugated goat anti-rabbit secondary antibody (1:200 dilution, Santa Cruz Biotechnology Inc, CA) at room temperature for 1 hour and the reactivity was visualized with peroxidase-substrate solution (diaminobenzidine, DAB) until the desired stain intensity developed. Sections were counterstained with Harris's hematoxylin for 35 seconds.

### RT-PCR and real-time PCR

Total RNA was extracted from the pellets of sorted SP and main population (MP, non-SP) cells using a Trizol Reagent (Invitrogen Corporation, Carlsbad, CA) and was reverse-transcribed into cDNA with a reverse transcription kit (Takara Bio Inc., Shiga, Japan) as per manufacturer instruction. The total RNA and cDNA of postpartum mouse uterus were prepared as the same way. β-actin was used as a housekeeping gene. PCR was performed using the following primers: β-actin primers, 5'-CCG TAA AGA CCT CTA TGC C-3' and 5'-CTC AGT AAC AGT CCG CCT A-3' for a 278-bp fragment; ESR1 primers, 5'-GCA CAG GAT GCT AGC CTT GTC TC-3' and 5'-CCA GCT TGC AGG TTC ATT GTG-3' for a 98-bp fragment. Cycling conditions for both β-actin and ESR1 were 94°C for 5 minutes, 94°C for 30 seconds, 56°C for 1 minute, and 72°C for 1 minute, 35 cycles. The PCR products were separated on 2% agarose gels for analysis.

Real-time PCR primers were the same for RT-PCR. Cycling conditions for β-Actin and ESR1 were 95°C for 10 seconds, 95°C for 5 seconds, 60°C for 31 seconds, 40 cycles. Melting curve analysis was performed to confirm the real-time qPCR products. Relative abundances of the target mRNAs were calculated using the 2-^ΔΔCT ^method [6].

### Western blot

The different postpartum days (Day1, 7, 14, 18, 21, 28) of mouse uteri were homogenated on ice with lysis buffer (7 mol/L urea, 2 mol/L thiourea, 4% [w/v] 3-[(3-cholamidopropyl)-dimethylammonio]-1-propane sulfonate (CHAPS), 2% [w/v] dithiothreitol (DTT), 2% [v/v] immobilized pH gradient [IPG] buffer, pH 3-10) in the presence of 1% (v/v) protease inhibitors cocktail kit (Pierce Biotechnology, Rockford, Illinois, USA). After centrifugation at 40000 g at 4°C for 1 hour, protein extracts in the supernatants were collected and stored at -80°C until use. Aliquots of 50 μg protein extracts from each sample were loaded and separated by 12% sodium dodecyl sulfate polyacrylamide gel electrophoresis (SDS-PAGE) and the resulting proteins were then transferred onto a nitrocellulose membrane. After treatment with blocking solution (5% non-fat milk powder in Tris-buffered saline [TBS; pH 7.4]) for 2 hours, the membranes were incubated with rabbit polyclonal antibodies of ESR1 (1:50, Santa Cruz Biotechnology, Santa Cruz, CA), or β-tubulin (1:5000, Abcam, Cambridge Science Park, Cambridge, UK) at 4°C overnight. The membrane was then incubated with HRP-conjugated anti-rabbit secondary antibodies (1:1000, Zhongshan Biotechnology Co. Ltd., Beijing, China) for 1 hour at 37°C, and examined by enhanced chemiluminescence (Amersham Biosciences, Uppsala, Sweden). The membranes were then scanned, and the signal intensity of each band was quantified using AlphaEaseFC (Fluorchem 5500) software (Alpha Innotech Corp., CA). Relative protein levels in each sample were normalized to the β-tubulin level in order to standardize the loading variations.

### Serum preparation and Hormone assays

Mice of different postpartum time (day1, 7, 14, 18, 21, 28) were anesthetized by 1% pentobarbital sodium and approximately 1 ml blood was withdrawn from the right ventricle. The blood samples were kept at room temperature for 2 hours, centrifuged at 1500 g for 10 minutes, and the sera were stored at -80°C until use. Concentrations of estradiol in the sera and conditioned medium of cultured SP cells were measured via a sensitive (<0.02 ng/mL) and reproducible (total coefficient of variation [CV], <10%) radioimmunoassay (RIA) with a measurement range from 0.1 to 20 ng/mL. The RIA kits were obtained from the Beifang Biotechnique Institute (Beijing, China).

### Clonogenic assay

SP cells were seeded in triplicate at 500 cells/cm^2 ^in flat-bottomed, 6-well culture plates. For the first 2 days, the culture medium was phenol red-free DMEM/F-12 (Gibco, USA) containing 5% FBS, 100 IU/ml penicillin, and 10 mg/ml streptomycin. Starting from Day 3, the cells were treated with different concentrations (10^-8 ^- 10^-6^M) of 17βE_2 _or 17βE_2 _in the presence of ICI182780 (inhibiter of ESR1, 10^-6^M), Cells were cultured for 15 days in 5% CO_2_: 95% air. The culture medium was changed every 2 days. Colonies were monitored microscopically daily to ensure that they were derived from single cells.

The dishes for clone analysis were fixed in 4% paraformaldehyde in PBS for 30 minutes, stained with Harris's hematoxylin for 5 minutes, then washed in running tap water for 20 minutes and dried. Clusters of cells were considered colonies when they were visible macroscopically and contained more than 50 cells. Colonies more than 70 cells were considered large clones. Colonies were counted and images were recorded. The cloning efficiency (CE) was determined from the formula: CE (%) = (number of colonies/number of cells seeded) × 100%.

### Cell proliferation and viability assays

The reduction of tetrazolium salts is now widely accepted as a reliable way to examine cell proliferation. The 3-(4,5-dimethylthiazoly1-2)-2,5-diphenyltetrazolium bromide(MTT) is reduced by metabolically active cells, in part by the action of dehydrogenase enzymes, to generate reducing equivalents such as NADH and NADPH. The resulting intracellular purple formazan can be solubilized and quantified by spectrophotometric means. Briefly, SP cells were plated at a density of 3 × 10^4^cells/well in 96-well plates, cultured for 2 days in phenol red-free serum medium and then exposed with different concentrations (10^-8 ^- 10^-6^M) of 17βE_2 _in the presence or absence of ICI182780 (10^-6^M) for 24 hours. The cells were then incubated with 0.5 mg/ml MTT for 4 hours at 37°C. The media was carefully removed and 150 μl of DMSO were added to each well for 15 minutes in order to solubilize the dark blue formazan crystals formed in intact cells. The absorbance was measured at 570 nm with the microplate reader (Bio-Tek ELX800, USA).

### Statistics

Each experiment was repeated at least three times. All data were presented as mean ± SD. One-way analysis of variance was used to compare the mRNA and protein levels. Chi-square analysis was used to compare the rates of SP cell large clones. A value of P < 0.05 was considered statistically significant.

## Results

### Variation of SP cells proportions along with postpartum days

**Figure 1:**
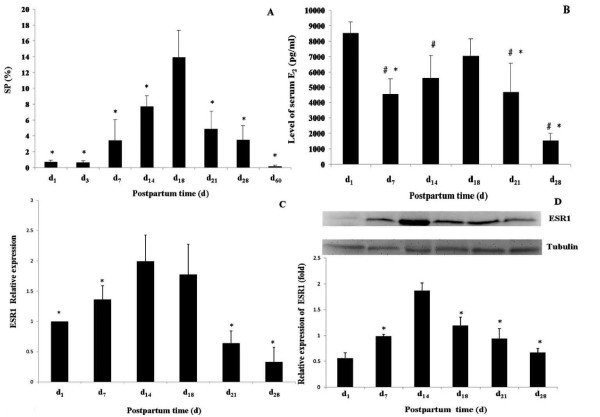
**Proportion of SP cells and ESR1expression in postpartum mouse uterus, as well as serum E_2 _level, in postpartum mice at different day**. Figure 1A. Proportion of SP cells in the uterus of postpartum mice at Day 1 to 60. *Compared with Day 18, P < 0.05. At Day 18, the proportion of SP cells was highest. Figure 1B. Levels of serum E_2 _in mice at different postpartum day. #compare to Day 1, P < 0.05; * compare to Day 18, P < 0.05. Levels of E_2 _in Day 1 and Day 18 were the highest. Figure 1C. Real-time RT-PCR analyses of ESR1mRNA expression in postpartum uterus. *compare to Day 14, P < 0.05. Expression of ESR1 mRNA in Day 14 was highest. Figure 1D. Western blot analyses of ESR1 expression in postpartum mouse uterus. ESR1 expression level at day1 was set as a standard as 1. *compare to Day 14, P < 0.05. Level of ESR1 protein in Day 14 was highest.

Fluorescence activated cell sorter (FACS) analysis showed that along with the changing of postpartum time (Day1, 3, 7, 14, 18, 21, 28, 60) the proportion of SP cells increased significantly and then gradually decreased after Day 18 (P < 0.05) (Figure 1A).

### Variation trend of serum E_2 _level along with postpartum days

Serum estradiol level was high at D1, and then remarkably decreased at Day 7 (Figure 1B). The E_2 _level, on the other hand, had a gradual increase from Day 7 to Day 18 after which it showed a decreasing trend (P < 0.05) (Figure 1B). This trend is representative of the change in SP cell proportion. These findings suggest that estradiol may play a role in the regulation of SP cells, which participate in the regeneration of postpartum mice endometrium.

### Expression of ESR1 in mouse endometrium at different postpartum days

Analysis with real-time PCR showed increased ESR1 expression within the first 14 days of postpartum, with the highest peak at Day 14 (P < 0.05) (Figure 1C). This trend was similar to the trend of SP cell ratio in the endometrium of postpartum mice. Western blot showed the same result as PCR analysis. Compared with other days of postpartum, ESR1 obviously expressed at highest level at Day 14 (P < 0.05) (Figure 1D).

**Figure 2:**
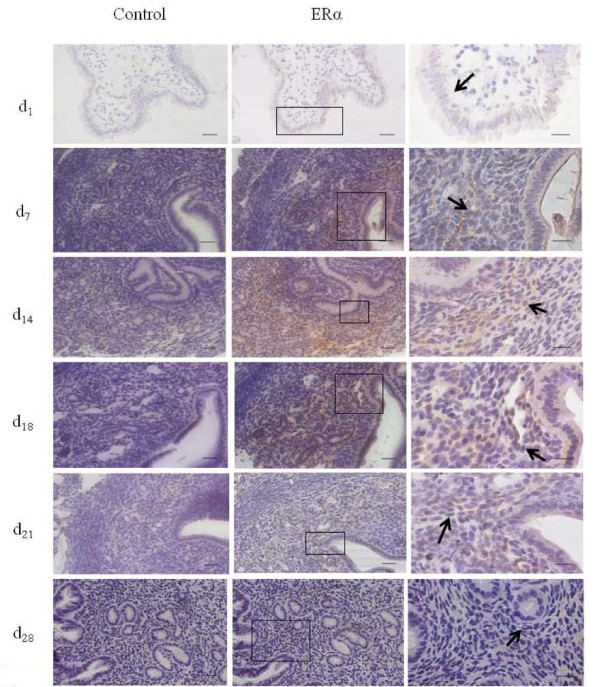
**Immunohistochemistry analyses of ESR1 expression in postpartum mouse uterus**. Left side was negative control, the middle and right showed ESR1 expression, which shown at middle (40×) and higher magnification (100×). Scale bar was 20 μm, while right side scale bar was 10 μm. The arrows indicate ESR1 immunopositive cells.

Immunohistochemistry showed the same variation as Real-time PCR analyses. Luminal epithelium at Day 1 expressed low level of ESR1, whereas stroma and glandular epithelium at Day 7 showed mid level of ESR1 expression. Stroma at Day 14 expressed high level of ESR1. From Day 18 to Day 28, expression of ESR1 decreased gradually. At Day 18, significant amounts of ESR1 were expressed in the Glandular epithelium. ESR1 expression was seen to decrease significantly at Day 21 and Day 28 (Figure 2).

### Preferential expression of ESR1 in SP cells when compared with non-SP cells

**Figure 3:**
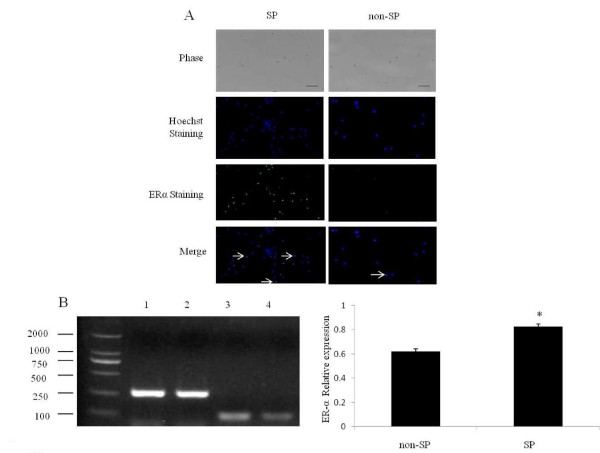
**Expression of ESR1 in SP and non-SP cells**. Figure 3A. Immunocytochemical analyses of ESR1 expression in SP and non-SP cells. Nuclear Hoechst staining (blue) and immunofluorescent labeling of ESR1 (green) were merged. Scale bar, 100 μm. The arrows indicate ESR1 immunopositive cells (Hoechst and ESR1 staining merged). Figure 3B, C. RT-PCR analyses of ESR1 expression in SP and non-SP cells. 1. SP Actin; 2. Non-SP Actin; 3. SP ESR1; 4. Non-SP ESR1. *P < 0.05.

We also examined the basal expression of ESR1 in SP cells and non-SP cells. RT-PCR analyses showed that the expression of ESR1 mRNA was higher in SP cells than that in non-SP cells (P < 0.05) (Figure 3B, C). Immunocytochemical analysis showed that 75.18 ± 5.47% of SP cells expressed ESR1, and only 32.28 ± 4.3% of non-SP cells expressed ESR1 (Figure 3A). These findings suggested that a large number of SP cells were able to respond to estrogen (target cells of estrogen).

### Cell proliferation of SP cells modulated by 17βE_2_

**Figure 4:**
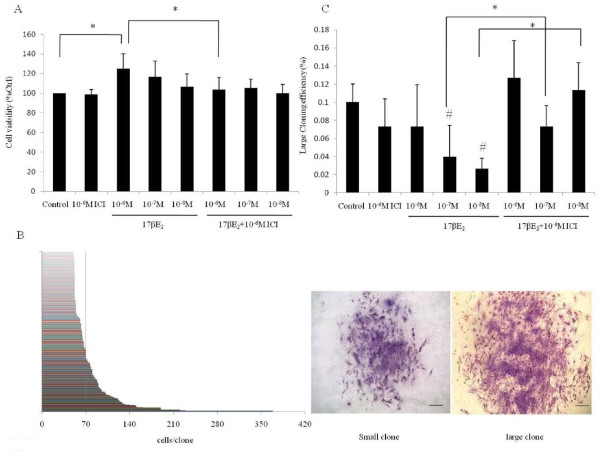
**Effect of 17β-E_2 _on the proliferation and formation of large cloning efficiency of SP cells**. Percentage and large cloning efficiency of SP cells treated with 17β-E_2 _and 17β-E_2 _+ICI182780 (Faslodex, inhibitor of ESR1) for 24 h. Figure 4A. SP cells were pretreated with 17β-E_2 _and 17β-E_2 _+ICI182780 at different concentration (10^-8 ^to10^-6^M) for 24 h, after which the cell viability was estimated by the MTT assay. *n *= 4 experiments. * Compared with control, p < 0.05. Figure 4B. Distribution of colony size of SP cells cultured in phenol red-free sera medium for 15 days. The number of cells per colony from 344 colonies was counted, with the data generated used to discriminate small and large colonies, as shown by the vertical line at 70 cells. Hematoxylin staining of small clone (left) and large clone (right). Scale bar, 100 μm. Figure 4C. Large cloning efficiency of SP cells treated with 17β-E_2 _and 17β-E_2 _+ICI182780 at different concentration (10^-8 ^to10^-6^M) for 15 days. #Compared to control, and *P < 0.05.

To study the proliferative effects of 17βE_2_, SP cells were treated with different concentrations (10^-8 ^- 10^-6^M) of 17βE_2 _in the presence or absence of ICI182780 (10^-6^M). 17βE_2 _at the dose of 10^-6 ^M induced a significant increase in SP cell numbers (P < 0.05), even though E_2 _at lower doses (10^-7^, 10^-8 ^M) could not significantly change SP cell numbers (P > 0.05) (Figure 4A). The stimulated proliferation was blocked by ICI182780 (P < 0.05) indicating the effect was specifically mediated through estrogen receptors.

### Variation of large CE of SP cells after 17βE_2 _treatment

Total CE of SP cells in all of 17βE_2 _treated groups did not significantly change. The CEs of large colonies were as follows: 0.073% ± 0.046% (n = 3) for 10^-6 ^M 17βE_2_, 0.04% ± 0.031% (n = 3)for 10^-7 ^M 17βE_2_, 0.027% ± 0.011%(n = 3) for 10^-8 ^M 17βE_2._, 0.126% ± 0.042 (n = 3) for 10^-6 ^M 17βE_2_+ICI182780, 0.073% ± 0.023% (N = 3) for 10^-7 ^M 17βE_2_+ICI182780, and 0.113% ± 0.03% (n = 3) for 10^-8 ^M 17βE_2_+ICI182780. There was a significant decrease in CE of large colonies of SP cells in groups treated with 10^-7 ^M and 10^-8 ^M of 17βE_2_. ICI182780 was seen to block this effect (P < 0.05) (Figure 4C). The size distribution of these SP cells colonies was also examined by scoring 344 colonies from nine samples and sorting them into numerical order. Figure 4B shows that colonies could be categorized into two groups: small colonies (<70 cells) or large colonies (>70 cells).

## Discussion

Adult stem cells have tissue-specific function as they are known to repair and maintain their corresponding tissues [3]. The concept that endometrial regeneration is mediated by endometrial stem/progenitor cells was proposed many years ago [7,8]. It is widely believed that endometrial restoration after menses suggests a need for estrogen-primed proliferation. In the previous study, it was shown that SP cells of postpartum murine endometrium were a heterogeneous population of endometrial stem/progenitor cells [3]. In the mouse, the normal estrus cycle recovers several days after the lactation is over, which is approximately 21-24 days after parturition. In this study, an initial increase was seen in the percentage of SP cells in postpartum murine endometrium. This increase was observed until Day 18, after which a gradual decrease was recorded. It was suggested that this phenotype might correlate with endocrinological hormonal changes after parturition. Uterus is an important target organ of estrogen. Estrogen receptor (ER) has two isoforms: ESR1 and ESR2. ESR1 is the main isoform of ER in humans and rodents. Although ESR1 and ESR2 were both expressed in endometrium, when compared with the wild type, it was seen that the uterus of the ESR1 knockout mice is immature with fewer glands. Even with the administration of exogenous E_2_, there was no thickening of the endometrium or the muscular layer in ESR1 knockout mice. This is indicative of the primary function of ESR1 in the uterus [9,10]. Estrogen regulates endometrial cell survival, viability and mitogenic activity via ESR1 [11,12]. Decreased expression of ESR2 has previously been seen in many estrogen-dependent tumors [13]. ESR2 acts as a modulator of ESR1 and has an anti-proliferative and pro-apoptotic role [14]. In this study, it was found that the expression of ESR1 was greater in SP cells, when compared with the expression in non-SP cells. Since ESR1 is the dominant isoform in the uterus, it was assumed that estrogen might regulate SP cells via the ESR1 function of regenerating the endometrium.

The E_2 _level in serum and the ESR1 expression in uterus showed the same trend with the proportion of SP cells and total uterus cells varying in different postpartum days (Day1 to Day 28). It was found that the E_2 _level in serum gradually increased from postpartum Day 7, and then declined after Day18. However, the ESR1 expression at both the gene level and the protein level peaked at postpartum Day 14. It was reported by Kang *et al. *[15] that after treatment with 17-ethinyl estradion (EE) (doses of 3.0 and 10.0 μg/kg/day), ESR1 expression decreased in the uterine luminal and glandular epithelium, as well as in the stroma and the uterine smooth muscle cells. The data suggested that the increased endogenous E_2 _level could negatively regulate ESR1 expression in uterus to some extent, which could have resulted in the discord of time-course of E_2 _level and ESR1 expression peaks.

In this study, it was found that ESR1 in mouse uterus at postpartum Day 14 was predominantly expressed in endometrium stroma, while it was highly expressed in glandular epithelium at postpartum Day 18. In previous studies, it was found that the ATP-binding cassette superfamily G member 2 (ABCG_2_) was mainly expressed in postpartum mouse endometrium stroma. ABCG_2 _was also expressed in a small part of vascular endothelial cells and glandular epithelium [3]. ABCG_2 _is specially expressed in SP cells. Estrogen promotes endometrium cell mitosis, which is an important factor for the development and functional maintenance of female reproductive system. In uterus, E_2 _promotes endothelial progenitor cell (EPC) differentiation, migration, proliferation, and apoptosis inhibition [16-18]. The enhanced biological activities in EPC by E_2 _are blocked by the specific ESR1 antagonist ICI182780, which indicates that the effect of estrogen on EPCs is via functional ESR1 in EPCs. In this study, we found that 10^-6^M E_2 _could significantly promote proliferation of SP cells in vitro and this effect could be blocked by ICI182780 (P < 0.05). Three other concentrations of E_2 _at a lower level showed concentration dependent tendency of stimulation of SP cell proliferation although they did not reach statistical significance. Therefore, E_2 _leads to the promotion of SP cells via ESR1 by participating in the regeneration of the endometrium.

Stem cells are able to differentiate, self-renew and replace themselves, into committed progenitors. These committed progenitors are differentiated transit amplifying (TA) cells, which rapidly proliferate and finally differentiate to produce a large number of terminally differentiated functional cells with no capacity for proliferation. It is possible that the large colonies are initiated by putative stem/progenitor cells and the small colonies are initiated by putative TA cells [2]. In this study, it was found that CE of large clones treated by 10^-7^M and 10^-8^M E_2 _notably decreased and this effect could be blocked by ICI182780 (P < 0.05). With E_2 _concentration gradually decreasing from 10^-6^M to 10^-8^M, CE of large clones also showed decreasing trend even though there was not a statistically significant difference recorded (P > 0.05). These results indicated that E_2 _at the circumambient concentration of physiological range could promote the committed differentiation of SP cells *in vitro*. The physiological serum concentration of 17βE_2 _in postpartum mice is about 10^-8 ^M. *Deasy et al.*, [17] when studying Duchenne Muscular Dystrophy (DMD), found that the female muscle-derived stem cells (MDSCs) regenerated skeletal muscle more efficiently than those of the males. It was therefore concluded that E_2 _at high concentrations promoted proliferation of SP cells of postpartum mouse endometrial, whereas E_2 _at physiological concentrations could promote differentiation of SP cells.

In clinical practice, many patients become infertile due to declining endometrial regeneration capability. The common reasons include abortion, infection and endocrinal dysfunction. Conversely, abnormal endometrial hyperplasia could lead to endometriosis, functional uterine bleeding and endometrial carcinoma [19-22]. The current study advanced the understanding of the role of SP cells in repairing postpartum endometrium and in the pathological mechanism of abnormal endometrial proliferation, as well as the accumulated materials for experimental and clinical applications of endometrial SP cells.

## Conclusions

In conclusion, evidence provided in the present study indicated that E_2 _promoted SP cells via ESR1, which in turn plays a role in the regeneration of the endometrium. It was observed that E_2 _at high concentrations promoted proliferation of SP cells of postpartum mouse endometrial, while E_2 _at physiological concentrations could lead to the promotion of differentiation of SP cells. Further studies will be required to elucidate the role of SP cells in repairing postpartum endometrium and in the pathological mechanism of abnormal endometrial proliferation.

## Competing interests

The authors declare that they have no competing interests.

## Authors' contributions

JX and FFH carried out the main experiments and wrote the first draft of manuscript. YGC and JYL, teachers of JX and FFH, designed the study and revised manuscript. YGC investigated data and proofread the final manuscript. JL, CYJ, LG XQQ and YDM participated in some experiments. All authors read and approved the final manuscript.
